# Housing Security and Settlement Intentions of Migrants in Urban China

**DOI:** 10.3390/ijerph19169780

**Published:** 2022-08-09

**Authors:** Zhen Wang, Mingzhi Hu, Yu Zhang, Zhuo Chen

**Affiliations:** 1 School of International and Public Affairs, Shanghai Jiao Tong University, Shanghai 200030, China; 2 School of Management, Chinese Academy of Housing and Real Estate, Zhejiang University of Technology, Hangzhou 310014, China; 3 School of Government Audit, Nanjing Audit University, Nanjing 211815, China

**Keywords:** housing security, settlement intentions, residence, migration

## Abstract

A huge gap remains in the urbanization rate between China and developed countries, although China has experienced a rapid growth in urbanization rate over the last decade. Critical to the future growth of urbanization is how to increase the settlement intentions of migrants. This study uses land supply for security housing as an exogeneous shock to the supply of security housing in the near future to explore how housing security is associated with intentions to settle down in destination cities of the migrant population. We found that increased land supply for security housing promotes the settlement intentions of migrants. Moreover, housing security is positively associated with permanent settlement intentions, while its relation to temporary settlement intentions is not significant. Lastly, the effect of housing security is larger for households with more housing expenditure. Our results have important theoretical and practical significance for the research on urban development and social welfare.

## 1. Introduction

The migrant population in China consists of the people who leave their hometowns without the official transfer of household registration (or hukou) from origin to destination, and who often have lower social status than local permanent residents [1]. Restrictions of the hukou system on labor mobility within China have been relaxed significantly since the 1990s [2]. At present, Chinese workers are free to move and work in any place within China [3]. Data from the Seventh National Census show that China’s migrant population reached 376 million in 2020, an increase of 69.73% compared with that in 2010. The massive influx of the migrant population to urban cities has contributed remarkably to the rapid economic growth in recent decades [4,5]. Regions have been competing to attract numerous migrant labor for sustainable economic growth, although an enormous migrant population could pose serious challenges to urban governance and sustainability, such as shortage of public resources, traffic congestion, and increased living costs [6,7]. 

China launched a series of security housing projects in the 1990s, which were a crucial part of the nationwide market-oriented economic reform in the country, to improve the housing conditions of low- and middle-income households [8,9]. At present, the Chinese government implements a people-oriented urbanization strategy by expanding the supply of security housing in urban areas in order to increase the urbanization rate to 65% by 2025. The determinants of the attraction of migrants are important and have been extensively examined in the literature [10,11,12,13,14]. Moreover, a significant issue for the settlement intentions of migrants involves housing conditions and living costs [9,15]. However, knowledge is limited on the extent to which housing security affects the settlement intentions of the migrant population.

This study uses the ratio of land supply area for security housing as a shock to security housing in the near future, particularly to explore how the future supply of security housing is associated with the intentions to settle down in the host cities of the migrant population and extends the literature in the following ways. First, we empirically examine the impact of housing security on the settlement intention of migrants, and find that increased land supply for security housing promotes the settlement intention of migrants. This finding is robust to variable measurement errors, error-term mis-specification, and omitted variable and sample selection biases. Second, we document the heterogeneity effect of housing security on the length of residence. Our results show that a high ratio of land supply area for security housing to the total land supply is associated with an increase in the permanent settlement intention. Meanwhile, the association between housing security and temporary settlement intention is not significant. Third, this study shows that the effect of housing security varies across households with different housing expenditure. The supply of security housing would decrease housing expenditure, thereby constituting the main component of household expenditures. We find that the effect of housing security is larger for households with more housing expenditure.

The remainder of this paper is organized as follows. Section 2 briefly introduces the security housing projects in China and the determinants of the settlement intentions of the migrant population. Section 3 introduces the data and variables. Section 4 reports the empirical results. Section 5 presents the discussion. Section 6 concludes the study.

## 2. Research Background and Literature Review

### 2.1. Security Housing Projects in China

The 1994–1998 housing reform that allowed state units to sell their public housing stock to their workers at heavily discounted prices and abolished the provision of welfare housing is an important milestone in China’s urban housing reform, which has resulted in a market-oriented urban housing provision system [16,17]. The housing market reform leads to a significant increase in homeownership rate, but it also causes a rapid increase in house prices [9,18]. With prices increasing in Chinese cities, housing has become substantially unaffordable, particularly for low- and moderate-income households [18,19]. To maintain sustainable urbanization, the central and local governments have introduced a series of security housing projects since the 1990s to improve the housing conditions of economically disadvantaged households. 

Security housing projects mainly include the Comfortable Housing Project (*anju gongcheng*), Economic and Comfortable Housing (*jingji shiyong fang*), Cheap Rental Housing (*lianzu fang*), and Public Rental Housing (*gongzu fang*). The housing built by the first two projects is, in principle, for sale and the housing built by the latter two projects is to rent out to qualified families. The Comfortable Housing Project was implemented in 1995 to provide affordable housing to low- and middle-income families and houses were required to be sold at construction costs [20]. This project has considerably improved the housing condition of urban households, although it has been blamed for causing numerous social problems, such as misappropriation of project funds and corruption [21]. The Economic and Comfortable Housing project has been promoted by the government since 1998. The size of economic and comfortable housing is limited to 60–80 square meters and its sale price is strictly controlled (profit margins are limited to under 3%) [22]. The provision of public rental housing was suspended after 1998, while the government allowed low-income households with a local hukou in the place of residence to apply for cheap rental housing and those without a local hukou to apply for public rental housing [23].

### 2.2. Determinants of the Migrant Population’s Settlement Intentions

Research on the settlement intentions of migrant populations involves a variety of research fields, including economics, sociology, environment, and mental health while the literature has demonstrated the complexity of settlement decisions. Neoclassical economics has argued that migration is driven by the benefits and costs involved [24]. Economic factors and geographical distance have been mostly examined and have been found to be critical to settlement decisions [25,26,27]. The majority of related studies have documented a positive effect of economic opportunities on migrant workers’ decision to settle down, and the impact of economic incentives decreases with an increase in the distance between destination and origin [15,28,29]. Several studies have emphasized the role of non-economic factors in settlement intentions, such as social networks and social stress [30,31,32,33]. Sociological theory suggests that migration could be considered a dynamic social process that depends primarily on sociocultural factors [10,34,35,36]. By strengthening social ties with local residents, migrants are likely to settle as they expand their access to information, improve their chances of homeownership, and accumulate social capital. 

Push–pull theory explains the decision to migrate in a similar manner [37]. For individuals or families, the push factors of migration mainly include local crime rates, the political situation, discrimination and housing conditions, and pull factors incorporate public services, facilities, and climate [28,37,38]. Rural migrants who have higher expectations of a convenient urban life, expect to provide better education for their children, and having relatives living in the same cities they are more willing to settle down [39]. Migrants who have social insurance have higher settlement intentions than those who have not [35], and they are more likely to settle down in cities with a large population and good climate conditions [35,40]. Previous studies have also investigated the impact of household demographic and socioeconomic characteristics on individuals’ settlement intentions. Overall, migrants who are employers or self-employed, younger, better educated, married, and those who live with a spouse are likely to settle down in the host cities [26,41]. Tang and Feng (2015) showed that the younger generation is more willing to settle down in cities compared with the older generation owing to differences in life stage and occupational experiences [42]. Li et al. (2020) examined how environmental quality is associated with settlement decisions, and they found that poor air quality reduces migrants’ settlement intentions, while the negative effect of air quality decreases as their income increases in the destination [43]. 

The Chinese government has retained extremely tight controls on migration across regions through the management of the hukou system, which was formally created in 1958 [44]. Each household in China holds either an urban or rural hukou based on birthplace, and rural hukou holders were forbidden to migrate into cities. Although hukou management has become loose over time, this process remains a salient feature of the Chinese labor market [45]. The migrant population is excluded from the public service and often discriminated against by their rural hukou status [30,46,47,48]. The hukou system has been regarded as a fundamental institutional barrier to the settlement intentions of migrants [48]. However, an increasing number of cities have relaxed the hukou conversion criteria, as proposed by the central government since 1997 [3]. Household registration restrictions have been completely removed in small- and medium-sized cities after the announcement of China’s new people-oriented urbanization plan in 2014. Instead, housing conditions have become an increasingly important factor related to migrants’ settlement intentions [15,48].

### 2.3. Security Housing and Migrant Population’s Settlement Intention

Housing costs account for a large proportion of the costs of living in urban cities, particularly in China, where housing prices have been soaring for the past decade [49]. According to data from the National Bureau of Statistics of China, housing expenditure accounts for nearly a quarter of the total household consumption expenditure in urban China. Homeownership is cherished in China because owning a home ensures a stable living space for the family, which is associated with emotional attachment and social belonging. Owning a home has even become a prerequisite for marriage in many regions in China [50,51]. Numerous studies have shown that soaring housing prices reduce the willingness of the migrant population to settle down by inflating the costs of living and making homeownership unaffordable [11,15,38,52,53]. 

We hypothesize that the provision of security housing would have a positive effect on the settlement intentions of the migrant population for two reasons. First, the supply of security housing, which is a quasi-public good provided by the government, improves the housing conditions and decreases housing costs. A large proportion of rural-to-urban migrants lives in urban villages with poor facilities and living environment [54,55,56,57]. China built nearly 40 million units of security housing from 2011 to 2015, and over 38 million people lived in security housing in China in 2019. The massive increase in the supply of low-rent security housing improves the housing quality and reduces the cost of living of the migrant population, thereby increasing their intention to settle down in destination cities. Second, the supply of low-cost security housing increases the probability of home buying among migrant population. The security housing project also helps cool down the housing market, making rents and housing considerably affordable [9].

## 3. Data and Identification Strategy

### 3.1. Data

Data for analysis are collected from two sources. City-level data of the land supply area for security housing and the total land supply area are obtained from the China Land Market Network (CLMN; http://www.landchina.com/, accessed on 15 March 2021) website. CLMN is operated by the Ministry of Land and Resources of China, which includes extensive information on all aspects of land supply in the country. We collected land transaction data, including total land area for security housing and total land area in 286 cities in 2011–2016 from CLMN. Data of other variables are obtained from the China Migrants Dynamic Survey (CMDS) implemented in 2017. CMDS is a nationwide household survey conducted by the National Health and Family Planning Commission of China. Migrants aged 15 years and above who have moved to the city for over one month from across different regions are randomly selected to be interviewed by the stratified sampling procedure.

### 3.2. Variables

The dependent variable in our analysis is an indicator variable of settlement intention in the host city, which is defined based on the following question in CMDS: “Do you plan to stay in the host city?” The independent variable is the average value of the ratio of the supply area for security housing over the past 5 years. We used several screenings for the sample. First, we restricted our attention to renters because security housing is only available for renters in the majority of cities. We define a person as a renter if he/she lives in rental housing when surveyed, including rented public housing provided by the government, market housing, work-unit housing, and other kinds of housing not owned by him/her (We identified renters based on the question “What kind of housing do you currently live in?”, which is the 308th question in the survey questionnaire). This strategy could cause sample selection bias if homeowners excluded in the sample are not randomly selected. We used the Heckman model to deal with this issue in the discussion of robustness checks. Second, we deleted rural observations because we are particularly interested in the settlement intention of migrants in urban cities, and there is nearly no housing market and housing security program in rural China. Third, observations with missing value were excluded. The final data set includes 49,396 observations in 233 cities. Table 1 shows the summary statistics of variables and the correlation matrix of variables. The majority of migrants intend to stay in the host city (over 80%). The land supply area is on average 669 hectare for the sample, and there is 26.7% of land supply area for security housing. As suggested in the literature (e.g., [28,42,58,59]), we considered household characteristics including gender, age, education, hukou status, marital status, political status, entrepreneur, self-employer, household size, and household income (Male is an indicator variable of people being male. Age refers to the age of individuals. Four-year college is an indicator variable of people with a four-year college education and above. Three-year college is an indicator variable of people with a three-year college education. Middle & high school is an indicator variable of people with middle or high school education. Urban hukou is an indicator variable of people with a non-agricultural hukou. Married is an indicator variable of people being married. Communist is an indicator of members in the Chinese Communist Party. Entrepreneur is an indicator variable of entrepreneurs. Self-employer is an indicator variable of self-employers. Household size refers to the number of family members. Income refers to the individual income per month (yuan). We acknowledge that due to data limitation we cannot control all factors which are significantly associated simultaneously with the settlement intention of migrants and the supply of housing security. We conduct a robustness check in Section 4.2.3 to ease the concern on omitted variable bias).

## 4. Empirical Results

### 4.1. Benchmark Regression Results

Given that the dependent variable in our analysis is a binary outcome of settlement intentions, we used the standard logit model to examine how the settlement intention is associated with housing security. The structure of the logit model has the latent variable format:

(1) $$\begin{array}{c} Stay_{ij}^{*}=\beta_{0,1}+\beta_{1,1}Ratio\;of\;land\;supply\;area\;for\;security\;housing_{ij}\\ +\beta_{2,1}Log{\left(Land\;supply\;area\right)}_{ij}+X_{ij}+\theta_{j}+\varepsilon_{ij}\;\;\; \end{array}$$

(2) $$Stay_{ij}=\{\begin{array}{c} 1,\;\;\;\;if\;Stay_{ij}^{*}>0\;\;\;\;\;\;\;\;\;\;\;\\ 0,\;\;\;\;Otherwise\;\;\;\;\;\;\;\;\;\;\;\;\;\;\; \end{array}$$

where $Stay_{ij}^{*}$ in the first equation, which is the latent variable equation, is a latent variable that can be written as a linear function of the covariates. $Stay_{ij}$ in the second equation, which is the choice equation, is an indicator variable of settlement intentions, which equals 1 if individual *i* in province *j* plans to stay in the host city, and 0 otherwise. $Ratio\;of\;land\;supply\;area\;for\;security\;housing_{ij}$ refers to the average value of the ratio of supply area for security housing over the past 5 years. $Log{\left(Land\;supply\;area\right)}_{ij}$ refers to the average value of the log value of the total land supply area in the past 5 years. $X_{ij}$ includes a vector of control variables, including gender, age, education, hukou status, marital status, political status, entrepreneur, self-employer, household size, and household income. $\theta_{j}$ stands for a complete set of province fixed effects. Lastly, $\varepsilon_{ij}$ denotes a random error term.

The results from the baseline regression are presented in Table 2. We estimated a series of different specifications by gradually increasing the number of controlled variables from left to right to see their effects on the settlement intention of migrants, which helps check the stability of the impact of housing security [60]. Column (1) reports the results of the simplest specification, which only controls the ratio of land supply area for security housing and residential land supply area measured at the city level. Column (2) further controls household characteristics, including gender, age, education, hukou status, marital status, political status, entrepreneur, self-employer, household size, and household income. We added the province dummies in the last column to control for the time-invariant regional characteristics.

Throughout columns (1) to (3), the coefficients of Ratio of land supply area for security housing and Log(Land supply area) remain positive and are statistically significant at the 1% level, suggesting that migrants are likely to stay with an increase in residential land supply (As a robustness check, we also added housing-related variables and city-level variables, including the log value of population density, the log value of GDP per capita, the ratio of the tertiary industry in GDP, and housing price to income ratio in the model. We do not control for population size since population size and ratio of land supply area for security is highly correlated (with a correlation coefficient over 0.8). The results after controlling for housing-related variables and city-level variables are reported in Appendix A, Table A1. Again, we can find that the coefficient of Ratio of land supply area for security housing remains positive and is statistically significant at the 1% level, suggesting that increased land supply for security housing promotes the settlement intention of migrants.). Moreover, increased land supply for security housing further promotes the settlement intention of migrants. Note that the estimates, including coefficients and marginal effects, are similar after the inclusion of the observed controls and unobservable regional characteristics, indicating that our results may be robust to omitted variables bias [60,61]. 

The impact of control variables on the settlement intentions of migrants is also interesting. As expected, migrants are less likely to stay in the host city where they currently live and work as they grow older. The reason is that the Chinese are likely to go back to the place where they grow up in their old age. The probability of staying in the local city increases with educational attainment and income because they are then capable of buying a home or apartment. Married people and households with large family sizes are likely to stay in the host city. The possible reason is that these households can integrate well into the city [62]. Interestingly, communists and entrepreneurs who have more resources and have higher social class are more willing to settle down in the host city where they currently work. 

### 4.2. Robust Checks

#### 4.2.1. Measurement Errors of Key Variables

Our estimates from the baseline regression may be biased if measurement errors exist on the dependent and independent variables. To ease this concern, this section uses alternative measurements to proxy for the dependent and independent variables. First, we proxy the settlement intention by the willingness to transfer to local hukou. The willingness to transfer to local hukou is an indicator variable, which equals 1 if migrants are willing to relocate their hukou in cities where they work, and equals 0 otherwise. We re-estimated the baseline regression model by replacing the dependent variable with the willingness to transfer to the local hukou. The results are reported in column (1) of Table 3. Note that the coefficient of Ratio of land supply area for security housing remains positive and statistically significant at the 1% level. 

Second, we substituted the average value of the ratio of land supply area for security housing over the past 5 years with that over the past 4 and 6 years. The estimated results, as shown in Columns (2) and (3) of Table 3, show a positive and significant coefficient of Ratio of land supply area for security housing. These findings suggest that previous results from baseline regressions are less likely to be driven by the measurement errors of key variables.

#### 4.2.2. Error-Term Mis-Specification

The error term is assumed to follow a logistical distribution in previous estimations. However, our previous estimations are inefficient if the error term actually follows a normal distribution. The best way to address this concern is to re-estimate the baseline regression by using the probit model.

The estimated results from the probit model are presented in Table 4. Note that an increased ratio of land supply area for security housing is significantly associated with a high likelihood of willingness to stay, holding the land supply area, the household characteristics, and the province-level characteristics as being equal. 

#### 4.2.3. Omitted Variables Bias

Another issue is that there may still exist a small amount of unobservable household and regional time-variant characteristics. These characteristics, which are significantly associated simultaneously with the settlement intention of migrants and supply of housing security, are omitted in the model. However, we controlled numerous observable household characteristics and unobservable time-invariant regional characteristics in previous estimations. To address the concern on the omitted variable bias, we adopted the test strategy developed by Oster (2019) [63], which estimates the unbiased treatment effect by considering the R-squared obtained in the regression that controls observable household characteristics and regional time-invariant characteristics (denoted by $\tilde{R}$). This test strategy builds on the theory of Altonji et al. (2005) [64]. This theory estimates omitted variable bias by observing the relative amount of selection on unobservables that would have to be relative to the amount of selection on observables to account for the entire treatment effect under the assumption that selection on observables is as important as selection on unobservables.

Suppose that $R_{max}$ indicates that the R-squared value of a theoretical specification includes observed and unobserved control variables. Oster (2019) suggests that $R_{max}=1.3\tilde{R}$ is sufficient for the calculation of a bias-adjusted treatment effect [63]. We assume the maximum of R-squared ($R_{max}$) to be 1.1, 1.2, 1.3, 1.4, and 1.5 times the R-squared from a fully controlled regression ($\tilde{R}$). The estimated results are reported in columns (3)–(7) of Table 5. Moreover, estimates in column (1) of Table 5 are from the linear regression that controls the ratio of land supply area for security housing and land supply area only. Column (2) of Table 5 further controls household characteristics and regional dummies. Note that the coefficient of Ratio of land supply area for security housing under different $R_{max}$ is between 0.0903 and 0.1641, which is above the controlled treatment effect (i.e., 0.0880). These findings suggest that the treatment effect from the baseline regression is unlikely to be severely influenced by the omitted variable bias.

#### 4.2.4. Sample Selection Bias

Sample observations are restricted to renters in previous estimations. This restriction aims to enhance the reliability of the observed influence of security housing on settlement intention. However, it may cause biased estimation results if renters are not randomly selected from the entire population. Consistent with previous studies that documented a positive effect of homeownership on settlement intention, our data show that homeowners are associated with a higher settlement intention in the host city (91.42% for homeowners vs. 80.44% for renters), and the difference is significant at the 1% level. We used the Heckman probit model, which includes homeowners and renters to ease this concern. The results are presented in Table 6. 

The coefficient of Ratio of land supply area for security housing remains positive and statistically significant at the 1% level, and its marginal effect is similar to the corresponding estimator reported in column (3) of Table 2. Furthermore, the p-value of the estimated correlation coefficient (ρ) is over 0.93, indicating that sample selection bias poses minimal threat to the baseline results.

### 4.3. Further Analyses

#### 4.3.1. Temporary Residence and Permanent Residence

We observe that housing security helps increase migrants’ settlement intention in the host cities. A further issue deserving to be explored would be the difference in the effect of housing security on the temporary (or short-term) and permanent (or long-term) settlement intention of migrants. The respondents surveyed in CMDS are queried on the length of their expected stay in the host cities if they plan to stay. Consistent with the literature on internal migrants in China [13,28], we considered the settlement residence intention of migrants is permanent if they plan to live in the host city for over 5 years.

We redefine the dependent variable of settlement intentions, which equals “1” if the respondent plans to live in the host city for not more than 5 years (temporary settlement intention), equals “2” if the respondent plans to live in the host city for more than 5 years (permanent settlement intention), and equals “0” otherwise. Table 7 shows the heterogeneous settlement residence intention associated with housing security by using the multinomial (polytomous) logistic regression model. Note that a high ratio of land supply area for security housing to the total land supply is associated with a significant propensity for permanent settlement intention, while the associations between housing security and temporary settlement intention is not significant. Unsurprisingly, housing security increases migrants’ permanent settlement intention rather than temporary settlement intention because housing security facilitate migrants’ social integration and self-identity in the host cities [65,66]. 

#### 4.3.2. Housing Security, Housing Expenditure, and Settlement Intentions

Housing price in urban China has been increasing since the 1994–1998 housing reform, in which public housing tenants were allowed to purchase their state-owned housing units at low prices [67]. Housing expenditure could also increase with increasing housing price because housing prices and rents are often positively correlated [68]. To the extent that housing prices are positively associated with renters, an increase in housing prices increases the financial stress for renters, potentially decreasing their settlement intention to the host cities. Therefore, we hypothesize that the effect of housing security is larger for households with more housing expenditure.

To test this hypothesis, we added the log value of housing expenditure and its interaction with the Ratio of land supply area for security housing in the baseline model. The results are presented in Table 8. As expected, the coefficient of the interaction term (Ratio of land supply area for security housing × Log(housing expenditure)) is positive and statistically significant at the 1% level, supporting our hypothesis. 

## 5. Discussion

This research used the supply of land for security housing as a shock to the supply of security housing in the near future, and explored how housing security is associated with intentions to settle down in the host cities of migrant population. We found that increased land supply for security housing promotes the settlement intention of migrants. This finding is robust with respect to the variable measurement errors, error-term mis-specification, omitted variable bias, and sample selection bias. 

The findings of this study carry meaningful policy implications. A huge gap exists in the urbanization rate between China and developed countries, although China has experienced a rapid growth rate in urbanization in the last decade. Data from the China National Bureau of Statistics show that urbanization rate reached 63.89% in 2020, 16.11% lower than the average of 80% in developed countries. China has also witnessed unprecedented rural-to-urban migration in recent years, which is a sharp contrast to previous decades when population movement was limited. How to increase the settlement intentions of migrants is critical to the future growth of urbanization. Our finding increases the need to incorporate housing security into policies that attract the settlement intentions of migrants to construct a harmonious society.

## 6. Conclusions

The massive transfer of labor from homeland rural areas to urban areas in China has made a remarkable contribution to the rapid increase in urbanization and economic growth in recent decades [4,5]. Data from the Seventh National Census show that China’s migrant population reached 376 million in 2020, accounting for 35% of the country’s population and increasing nearly 70% over the past decade. Local cities have implemented various policies to attract migrants. China launched a series of security housing projects in the 1990s that provided low-rent or low-cost housing for qualified households who generally had a low income and lived in a poor housing condition [8,9]. On average, the migrant population has low income, and the majority lives in urban villages with housing difficulties [54,55,56,57]. The provision of security housing would have a positive effect on the settlement intentions of migrants.

By combining city-level data of land supply area for security housing from CLMN and household-level data of settlement intentions of migrants from CMDS, this paper empirically examined the association between housing security and settlement intentions of migrants. This exploration has important theoretical and practical significance for the research on urban development and social welfare. This study has also several limitations. First, this study measured housing security at the city level. Hence, the results may be biased if the sample surveyed is not randomly selected from the full population. This concern is mitigated in this research because CMDS is a large-scale national survey on migrant population covering all provinces in mainland China, with a sample size of nearly 200,000 households. In addition, we only considered the cross-sectional relationship between housing security and settlement intentions. The housing and labor markets have changed substantially over the last decades. An interesting aspect would be to explore whether the effect of housing security changes correspondingly with housing market conditions. 

## Figures and Tables

**Table 1:** Summary statistics and correlation matrix.

Variable	Obs	Mean	Std.Dev.	1	2	3	4	5	6
1. Stay	49,396	0.804	0.397	1.000					
2. Ratio of land supply area for security housing	49,396	0.267	0.131	0.027 ***	1.000				
3. Land supply area	49,396	669.0	601.7	0.023 ***	−0.111 ***	1.000			
4. Male	49,396	0.556	0.497	0.003	0.014 ***	−0.005	1.000		
5. Age	49,396	35.41	10.04	−0.016 ***	0.021 ***	−0.026 ***	0.127 ***	1.000	
6. Four-year college	49,396	0.065	0.247	0.049 ***	0.012 ***	0.047 ***	−0.023 ***	−0.146 ***	1.000
7. Three-year college	49,396	0.107	0.310	0.037 ***	0.005	0.049 ***	−0.036 ***	−0.201 ***	−0.092 ***
8. Middle & high school	49,396	0.686	0.464	−0.017 ***	−0.024 ***	−0.008 *	0.089 ***	−0.048 ***	−0.390 ***
9. Urban hukou	49,396	0.138	0.345	0.021 ***	0.017 ***	0.038 ***	−0.010 **	−0.007	0.264 ***
10. Married	49,396	0.764	0.425	0.065 ***	0.087 ***	−0.063 ***	0.047 ***	0.453 ***	−0.118 ***
11. Communist	49,396	0.042	0.201	0.033 ***	0.004	0.008 *	0.042 ***	0.007	0.232 ***
12. Entrepreneur	49,396	0.052	0.222	0.037 ***	0.010 **	−0.016 ***	0.038 ***	0.017 ***	−0.019 ***
13. Self-employer	49,396	0.354	0.478	−0.014 ***	−0.008 *	−0.087 ***	0.011 **	0.194 ***	−0.156 ***
14. Household size	49,396	2.991	1.233	0.063 ***	0.044 ***	−0.104 ***	0.037 ***	0.247 ***	−0.142 ***
15. Income	49,396	4241	3456	0.074 ***	0.049 ***	0.047 ***	0.156 ***	−0.051 ***	0.156 ***
	**7**	**8**	**9**	**10**	**11**	**12**	**13**	**14**	**15**
7. Three-year college	1.000								
8. Middle & high school	−0.512 ***	1.000							
9. Urban hukou	0.171 ***	−0.161 ***	1.000						
10. Married	−0.147 ***	0.051 ***	−0.068 ***	1.000					
11. Communist	0.072 ***	−0.125 ***	0.107 ***	−0.001	1.000				
12. Entrepreneur	0.000	0.029 ***	0.026 ***	0.074 ***	0.003	1.000			
13. Self-employer	−0.141 ***	0.100 ***	−0.111 ***	0.252 ***	−0.063 ***	−0.174 ***	1.000		
14. Household size	−0.147 ***	0.077 ***	−0.129 ***	0.613 ***	−0.025 ***	0.064 ***	0.235 ***	1.000	
15. Income	0.063 ***	−0.039 ***	0.114 ***	0.079 ***	0.061 ***	0.238 ***	0.004	0.043 ***	1.000

Note: “***”, “**”, and “*” denote significance at 1%, 5%, and 10% level, respectively. Data of *Ratio of* land supply area for security housing and Land supply area are from the China Land Market Network website (http://www.landchina.com/, accessed on 15 march 2021). Data of other variables are from the CMDS 2017. We calculated the Variance Inflation Factors (VIFs) of all variables based on an Ordinary Least Squares regression to test for multicollinearity. The maximum value of VIFs obtained in the model is 2.71 (Ratio of land supply area for security housing), and the mean value is 2.02, well below the rule-of-thumb cutoff of 10 for regression models.

**Table 2:** Estimated results of housing security and settlement intentions (Logit regression).

	(1)		(2)		(3)	
	Coef.	Marginal Effect	Coef.	Marginal Effect	Coef.	Marginal Effect
Ratio of land supply area for security housing	0.567 ***	0.089 ***	0.378 ***	0.058 ***	0.568 ***	0.086 ***
	(0.087)	(0.014)	(0.089)	(0.014)	(0.145)	(0.022)
Log(Land supply area)	0.102 ***	0.016 ***	0.093 ***	0.014 ***	0.102 ***	0.016 ***
	(0.013)	(0.002)	(0.013)	(0.002)	(0.020)	(0.003)
Male			−0.054 **	−0.008 **	−0.038	−0.006
			(0.024)	(0.004)	(0.024)	(0.004)
Age			−0.005 ***	−0.001 ***	−0.006 ***	−0.001 ***
			(0.001)	(0.000)	(0.001)	(0.000)
Four-year college			0.748 ***	0.115 ***	0.707 ***	0.108 ***
			(0.069)	(0.011)	(0.070)	(0.011)
Three-year college			0.542 ***	0.084 ***	0.555 ***	0.084 ***
			(0.053)	(0.008)	(0.054)	(0.008)
Middle & high school			0.194 ***	0.030 ***	0.214 ***	0.033 ***
			(0.034)	(0.005)	(0.035)	(0.005)
Urban hukou			−0.012	−0.002	−0.008	−0.001
			(0.037)	(0.006)	(0.037)	(0.006)
Married			0.317 ***	0.049 ***	0.274 ***	0.042 ***
			(0.037)	(0.006)	(0.037)	(0.006)
Communist			0.249 ***	0.038 ***	0.253 ***	0.038 ***
			(0.068)	(0.010)	(0.069)	(0.010)
Entrepreneur			0.270 ***	0.042 ***	0.324 ***	0.049 ***
			(0.061)	(0.009)	(0.062)	(0.009)
Self-employer			−0.072 ***	−0.011 ***	0.006	0.001
			(0.026)	(0.004)	(0.027)	(0.004)
Household size			0.111 ***	0.017 ***	0.113 ***	0.017 ***
			(0.013)	(0.002)	(0.013)	(0.002)
Log(Income)			0.249 ***	0.038 ***	0.172 ***	0.026 ***
			(0.021)	(0.003)	(0.022)	(0.003)
Other variables controlled						
Province dummies	No		No		Yes	
Pseudo R-squared	0.0019		0.0206		0.0348	
Observations	49,396		49,396		49,396	

Note: “***” and “**” denote significance at 1% and 5% level, respectively; standard errors are reported in parentheses; data of Ratio of land supply area for security housing and Land supply area are from the CLMN; data of other variables are from the CMDS 2017.

**Table 3:** Estimated results of housing security and settlement intentions (Alternative definitions of variables).

	(1) Transfer to Local Hukou		(2) Settlement Intentions		(3) Settlement Intentions	
	Coef.	Marginal Effect	Coef.	Marginal Effect	Coef.	Marginal Effect
**The average value in past 5 years**						
Ratio of land supply area for security housing	1.203 ***	0.250 ***				
	(0.124)	(0.026)				
Log(Land supply area)	0.239 ***	0.050 ***				
	(0.018)	(0.004)				
**The average value in past 4 years**						
Ratio of land supply area for security housing			0.394 ***	0.060 ***		
			(0.133)	(0.020)		
Log(Land supply area)			0.094 ***	0.014 ***		
			(0.019)	(0.003)		
**The average value in past 6 years**						
Ratio of land supply area for security housing					0.744 ***	0.113 ***
					(0.148)	(0.022)
Log(Land supply area)					0.102 ***	0.015 ***
					(0.020)	(0.003)
Other variables controlled						
Household characteristics	Yes		Yes		Yes	
Province dummies	Yes		Yes		Yes	
Pseudo R-squared	0.0925		0.0345		0.0350	
Observations	49,396		49,396		49,396	

Note: “***” denotes significance at 1% level; standard errors are reported in parentheses; Household characteristics include gender, age, education, hukou status, marital status, political status, entrepreneur, self-employer, household size and household income; data of Ratio of land supply area for security housing and Land supply area are from the CLMN; data of other variables are from the CMDS 2017.

**Table 4:** Estimated results of housing security and settlement intentions (Probit regression).

	Coef.	Marginal Effect
Ratio of land supply area for security housing	0.325 ***	0.087 ***
	(0.082)	(0.022)
Log(Land supply area)	0.058 ***	0.016 ***
	(0.011)	(0.003)
Other variables controlled		
Household characteristics	Yes	
Province dummies	Yes	
Pseudo R-squared	0.0347	
Observations	49,396	

Note: “***” denotes significance at 1% level; Standard errors are reported in parentheses; Household characteristics include gender, age, education, hukou status, marital status, political status, entrepreneur, self-employer, household size and household income; Data of Ratio of land supply area for security housing and Land supply area are from the CLMN; Data of other variables are from the CMDS 2017.

**Table 5:** Coefficient robustness to unobservable selection bias based on Oster (2019).

	*Baseline Effect*	*Controlled Effect*	Bias-Adjusted *β*				
			$R_{\max}=1.1\tilde{R}$	$R_{\max}=1.2\tilde{R}$	$R_{\max}=1.3\tilde{R}$	$R_{\max}=1.4\tilde{R}$	$R_{\max}=1.5\tilde{R}$
Ratio of land supply area for security housing	0.0889 ***	0.0880 ***	0.0903	0.0940	0.1002	0.1138	0.1641
	(0.0136)	(0.0232)					
Log(Land supply area)	0.0163 ***	0.0181 ***					
	(0.0021)	(0.0033)					
Observations	49,396	49,396	49,396	49,396	49,396	49,396	49,396
R-squared	0.0019	0.0328	0.03608	0.03936	0.04264	0.04592	0.0492

Notes: “***” denotes significance at 1% level; Estimates in column (1) are from the linear regression that controls the ratio of land supply area for security housing and the land supply area only; column (2) further controls household characteristics, including gender, age, education, hukou status, marital status, political status, entrepreneur, self-employer, household size and household income, and regional dummies; standard errors are reported in parentheses; data of Ratio of land supply area for security housing and Land supply area are from the CLMN; data of other variables are from the CMDS 2017.

**Table 6:** Estimated results of housing security and settlement intention (Heckman probit model).

	Coef.	Marginal Effect
Ratio of land supply area for security housing	0.784 ***	0.127
	(0.159)	(0.080)
Log(Land supply area)	0.065 ***	0.011
	(0.023)	(0.007)
ρ	0.029	
	(0.357)	
LR test of indep. eqns.		
Chi-square	0.010	
Prob > Chi-square	0.9339	
Other variables controlled		
Household characteristics	Yes	
Province dummies	Yes	
Wald chi-sauqre	554.67 ***	
Observations	69,532	

Note: “***” denotes significance at 1% level; Standard errors are reported in parentheses; household characteristics include gender, age, education, hukou status, marital status, political status, entrepreneur, self-employer, household size and household income; data of Ratio of land supply area for security housing and Land supply area are from the CLMN; data of other variables are from the CMDS 2017.

**Table 7:** Estimated results of housing security and settlement intention (Temporary residence vs. permanent residence).

	(1) Temporary Residence		(2) Permanent Residence	
	Coef.	Marginal Effect	Coef.	Marginal Effect
Ratio of land supply area for security housing	−0.1088	−0.072	0.7048 ***	0.1553
	(0.1441)		(0.1362)	
Log(Land supply area)	0.0321	0.0030	0.1150 ***	0.0216
	(0.0205)		(0.0193)	
Other variables controlled				
Household characteristics	Yes		Yes	
Province dummies	Yes		Yes	
Pseudo R-squared	0.0408		0.0408	
Observations	49,396		49,396	

Note: “***” denotes significance at 1% level; standard errors are reported in parentheses; household characteristics include gender, age, education, hukou status, marital status, political status, entrepreneur, self-employer, household size and household income; data of Ratio of land supply area for security housing and Land supply area are from the CLMN; data of other variables are from the CMDS 2017.

**Table 8:** Estimated results of housing security, housing expenditure and settlement intentions.

	Coef.	Marginal Effect
Ratio of land supply area for security housing × Log(housing expenditure)	0.101 ***	0.015 ***
	(0.034)	(0.005)
Log(housing expenditure)	0.003	0.000
	(0.010)	(0.002)
Ratio of land supply area for security housing	0.043	0.006
	(0.230)	(0.035)
Log(Land supply area)	0.096 ***	0.015 ***
	(0.020)	(0.003)
Other variables controlled		
Household characteristics	Yes	
Province dummies	Yes	
Pseudo R-squared	0.0357	
Observations	49,396	

Note: “***” denotes significance at 1% level; Standard errors are reported in parentheses; household characteristics include gender, age, education, hukou status, marital status, political status, entrepreneur, self-employer, household size and household income; data of Ratio of land supply area for security housing and Land supply area are from the CLMN; data of other variables are from the CMDS 2017.

## Data Availability

Data sharing is not applicable to this article.

## Appendix A

**Table A1:** Estimated results of housing security and settlement intentions (Logit regression).

	(1)	
	Coef.	Marginal Effect
Ratio of land supply area for security housing	0.387 ***	0.059 ***
	(0.148)	(0.023)
Log(Land supply area)	0.025 ***	0.004 ***
	(0.007)	(0.001)
Male	−0.023	−0.003
	(0.025)	(0.004)
Age	−0.006 ***	−0.001 ***
	(0.001)	(0.000)
Four-year college	0.683 ***	0.104 ***
	(0.073)	(0.011)
Three-year college	0.537 ***	0.082 ***
	(0.057)	(0.009)
Middle & high school	0.200 ***	0.030 ***
	(0.036)	(0.005)
Urban hukou	0.015	0.002
	(0.040)	(0.006)
Married	0.244 ***	0.037 ***
	(0.040)	(0.006)
Communist	0.239 ***	0.036 ***
	(0.071)	(0.011)
Entrepreneur	0.371 ***	0.057 ***
	(0.065)	(0.010)
Self-employer	0.020	0.003
	(0.028)	(0.004)
Household size	0.102 ***	0.016 ***
	(0.013)	(0.002)
Log(Income)	0.117 ***	0.018 ***
	(0.024)	(0.004)
Log(Population density)	0.051 **	0.008 **
	(0.021)	(0.003)
Log(GDP per capita)	0.292 ***	0.044 ***
	(0.043)	(0.007)
Ratio of the tertiary industry in GDP	0.008 ***	0.001 ***
	(0.002)	(0.000)
Housing price to income ratio	−0.049 ***	−0.008 ***
	(0.012)	(0.002)
Other variables controlled		
Province dummies	Yes	
Pseudo R-squared	0.0366	
Observations	45,765	

Note: “***” and “**” denote significance at 1% and 5% level, respectively; standard errors are reported in parentheses; data of Ratio of land supply area for security housing and Land supply area are from the CLMN; data of other variables are from the CMDS 2017.
